# Boron Nanoparticle-Enhanced Proton Therapy: Molecular Mechanisms of Tumor Cell Sensitization

**DOI:** 10.3390/molecules29163936

**Published:** 2024-08-21

**Authors:** Anton L. Popov, Danil D. Kolmanovich, Nikita N. Chukavin, Ivan V. Zelepukin, Gleb V. Tikhonowski, Andrei I. Pastukhov, Anton A. Popov, Alexander E. Shemyakov, Sergey M. Klimentov, Vladimir A. Ryabov, Sergey M. Deyev, Irina N. Zavestovskaya, Andrei V. Kabashin

**Affiliations:** 1 P. N. Lebedev Physical Institute of the Russian Academy of Sciences, Leninsky Prospect 53, Moscow 119991, Russia; a.popov@lebedev.ru (A.L.P.); kdd100996@mail.ru (D.D.K.); shemyakovae@lebedev.ru (A.E.S.); ryabov@lebedev.ru (V.A.R.); 2 Institute of Theoretical and Experimental Biophysics, Russian Academy of Sciences, 3 Institutskaya St., Pushchino 142290, Russia; chukavinnik@gmail.com; 3 Shemyakin-Ovchinnikov Institute of Bioorganic Chemistry, Russian Academy of Sciences, Moscow 117997, Russia; ivan.zelepukin@gmail.com (I.V.Z.); biomem@mail.ru (S.M.D.); 4 Department of Medicinal Chemistry, Uppsala University, 75310 Uppsala, Sweden; 5 Institute of Engineering Physics for Biomedicine (PhysBio), National Research Nuclear University MEPhI, Kashirskoe Shosse 31, Moscow 115409, Russia; gtikhonowski@gmail.com (G.V.T.); aapopov1@mephi.ru (A.A.P.); smklimentov@mephi.ru (S.M.K.); 6 LP3, CNRS, Aix-Marseille University, 13288 Marseille, France; andrei.pastukhov@etu.univ-amu.fr; 7 National Research Center “Kurchatov Institute”, Academician Kurchatov Square 1, Moscow 123182, Russia; 8 “Biomarker” Research Laboratory, Institute of Fundamental Medicine and Biology, Kazan Federal University, 18 Kremlyovskaya St., Kazan 420008, Russia; 9 Institute of Molecular Theranostics, Sechenov University, Moscow 119991, Russia

**Keywords:** pulsed laser ablation in liquids, proton therapy, boron nanoparticles, proton–boron capture therapy, nanoparticle-enhanced proton therapy

## Abstract

Boron-enhanced proton therapy has recently appeared as a promising approach to increase the efficiency of proton therapy on tumor cells, and this modality can further be improved by the use of boron nanoparticles (B NPs) as local sensitizers to achieve enhanced and targeted therapeutic outcomes. However, the mechanisms of tumor cell elimination under boron-enhanced proton therapy still require clarification. Here, we explore possible molecular mechanisms responsible for the enhancement of therapeutic outcomes under boron NP-enhanced proton therapy. Spherical B NPs with a mode size of 25 nm were prepared by methods of pulsed laser ablation in water, followed by their coating by polyethylene glycol to improve their colloidal stability in buffers. Then, we assessed the efficiency of B NPs as sensitizers of cancer cell killing under irradiation with a 160.5 MeV proton beam. Our experiments showed that the combined effect of B NPs and proton irradiation induces an increased level of superoxide anion radical generation, which leads to the depolarization of mitochondria, a drop in their membrane mitochondrial potential, and the development of apoptosis. A comprehensive gene expression analysis (via RT-PCR) confirmed increased overexpression of 52 genes (out of 87 studied) involved in the cell redox status and oxidative stress, compared to 12 genes in the cells irradiated without B NPs. Other possible mechanisms responsible for the B NPs-induced radiosensitizing effect, including one related to the generation of alpha particles, are discussed. The obtained results give a better insight into the processes involved in the boron-induced enhancement of proton therapy and enable one to optimize parameters of proton therapy in order to maximize therapeutic outcomes.

## 1. Introduction

Proton beam therapy is considered one of the most promising and effective types of therapy for radioresistant tumors and tumors localized near vital organs [1]. The key advantage of proton therapy lies in the possibility of targeted irradiation of the pathological focus due to the absorption of a maximal dose in the Bragg peak, which is located at the end of the particle range, and the depth of its location depends on the proton energy [2]. However, due to particular scattering characteristics, proton therapy does not render possible high accuracy of dose distribution planning [3]. Variations in the patient’s anatomy along the path of a proton beam during the therapy procedure can disturb the conformality of dose distribution [4], which in turn can lead to a significant deviation in dose distribution from the biologically effective one.

A promising way in the improvement in proton therapy modality is related to the involvement of proton sensitizers to enhance the efficiency of the therapy’s outcome. Here, the most popular approach is related to the use of metal nanoparticles (NPs) of high-atomic-number (Z) elements such as Au [5,6,7], Fe [8], Pt [9], and Bi [10] to enhance the therapeutic effect. In particular, Kim et al. [6] reported a remarkable improvement in mice survival treated with 40 MeV protons with some tumor regression at a 100–300 mg Au/kg NP dose. The mechanism of therapeutic enhancement in the case of metal NPs is mostly attributed to a proton beam-induced generation of reactive oxygen species (ROS), which appear via the radiolysis of NP-surrounding water by Auger electrons emitted from the NPs [11] or catalytic processes occurring at the NP/water interface [12,13]. Such ROS are known to efficiently destroy cancer cells [14] to enable a prominent enhancement in proton therapy efficacy [15,16].

Another appealing sensitizing pathway for proton therapy enhancement is related to the use of boron (B)-based compounds. Cirrone G. et al. explored borocaptate sodium (BSH) solutions with B at concentrations of 40 and 80 ppm as sensitizers of proton therapy enhancement [17] and recorded a remarkable improvement in cytogenetic effect on prostate cancer DU145 cells irradiated with a 62 MeV proton beam. The authors recorded a significant increase in the number of chromosomal aberrations after the irradiation in the presence of BSH. Later, the same group provided results of the first preclinical evaluation of boron-containing BSH in an in vivo glioblastoma model [18]. The authors used real-time µ-positron emission tomography/computed tomography (µPET-CT) to report a significant increase in the therapeutic efficacy of boron-enhanced proton therapy compared to conventional geometry, inducing increased cell death and mitophagy, which confirms the efficiency of this modality.

Despite the demonstrated effect, the mechanism of boron-enhanced proton therapy still remains disputable. Cirrone, Cammarata et al. [17,18] suggested that the enhanced proton therapy action can be due to a nuclear mechanism, leading to the generation of α-particles with a high linear energy transfer (LET), as it was earlier proposed and considered theoretically by Yoon, Jung et al. [19,20,21]. This mechanism implies the participation of ^11^B isotopes from boron-based compounds in a nuclear reaction p + ^11^B → 3α (p-B), which is supposed to have a resonant maximum for proton energy of 675 keV [22]. In turn, α-particles are known to initiate unrepairable destruction of cancer cell DNAs, similar to how it happens in the case of boron–neutron capture therapy (BNCT) [23,24]. Denoting such a boron-enhanced proton modality as proton–boron capture therapy (PBCT), Cirrone G., Cammarata F., et al. expected a high precision in cancer cell elimination, profiting from a short path length of α-particles in water (less than 20 microns), which is comparable with the cell’s size [17,18]. However, alternative studies do not always confirm the reported high efficiency of this approach and even doubt the possibility of generation of α-particles under these irradiation conditions [13,25,26]. While confirming the presence of the radiosensitizing effect in the boron–proton interaction, the authors claimed that a similar efficiency could be achieved with X-ray irradiation, which makes the presence of nuclear reactions in such an interaction quite unlikely. The authors suggested that the identified effects of radiosensitization may strongly depend on the type of cells, the conditions of their cultivation and treatment with a boron-containing drug, as well as on their specific boron metabolism or the bystander effect [25]. Alternative nuclear mechanisms of boron-based proton sensitization can be considered within a “ternary” model [26], describing possible alternative nuclear reactions, including nuclear reactions between thermal neutrons (as a “by-product” of proton-target interaction) and ^10^B, which comprises 20% of naturally abundant boron, but the cross sections of these reactions do not seem to be consistent with the observed effects.

Non-nuclear mechanisms for boron-enhanced proton therapy may also not be ruled out, either. As an example, after the ionization of boron nuclei, one can expect the formation of Auger and secondary electrons, which could generate active radicals as well as cause radiolysis of water and other chemical reactions. All these phenomena could be responsible for toxic effects on cancer cells, as is the case with metal NPs. These non-nuclear effects can be enhanced by catalytic processes on the surface of nanoparticles [13]. Therefore, further investigation of the molecular mechanisms of increasing the efficiency of proton radiation in the presence of boron-containing drugs is complex and non-trivial, but it presents a very urgent and important task. 

Profiting from approaches of nanotechnology and newly emerging nanoradiopharmaceuticals [27], we recently introduced the concept of boron nanoparticle-enhanced proton therapy [28], which implied the use of elemental boron (B) nanoparticles (NPs) as sensitizers for proton therapy enhancement (instead of BSH). Prepared by methods of femtosecond-pulsed laser ablation in water [29], B NPs had exceptional purity and were coated with polyethylene glycol (PEG) to improve their colloidal stability and biocompatibility. We showed that the irradiation of MNNG/Hos cells at a dose of 3 Gy in the presence of 80 and 100 ppm of B NPs led to a drastic decrease in the number of formed cell colonies compared to the control samples, evidencing a strong proton therapy enhancement effect mediated by B NPs. The advantage of using boron NPs compared to boron phenylalanine (BPA) or BSH is the possibility of their more efficient delivery of boron and its accumulation in cancer cells, profiting from the EPR effect or using specially designed targeting molecules. In addition, one can expect the involvement of additional functionalities based on the intrinsic properties of nanomaterials (e.g., photothermal therapy [29]) to achieve synergetic enhancement effects. However, the molecular mechanisms responsible for the enhancement of therapeutic outcomes under boron NP-enhanced proton therapy are still not clear. In particular, our data showed that the proton beam irradiation of B NPs leads to the generation of reactive oxygen species (ROS), as identified by using the fluorescence of CellROX dye, which evidences a possible involvement of a non-nuclear mechanism of cancer cell kill-related oxidative stress. 

Here, we continue the investigation of boron nanoparticle-enhanced proton therapy; namely, we explore possible molecular mechanisms responsible for the enhancement of therapeutic outcomes.

## 2. Results

The efficiency of boron–proton interactions is directly related to the local boron content in the cells. Intracellular delivery of boron using NPs is capable of increasing this efficiency as well as providing locally higher concentrations compared to boron-containing drugs (BPA or BSH). 

**Figure 1:**
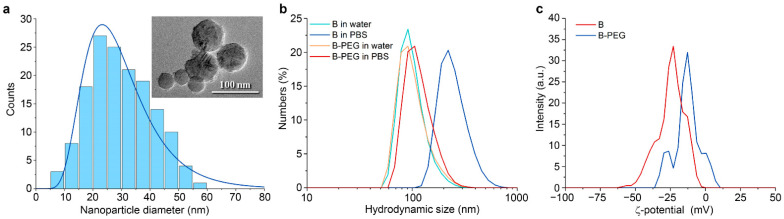
(**a**) Analysis of B NP size distribution with transmission electron microscopy. (**b**) Hydrodynamic size distributions of B NPs and B-PEG NPs after 5 min of incubation in water (pH 6) and PBS buffer (pH 7.4). (**c**) Zeta-potential distributions of B NPs and B-PEG NPs in 10 mM NaCl solution (pH 6).

Here, we synthesized B nanoparticles by the method of pulsed laser ablation in liquids, as described before [29]. Transmission electron microscopy (Figure 1a) has shown that nanoparticles were spherical with a lognormal size distribution. The mode value ± the half-width of the peak was 25 ± 12 nm. The hydrodynamic diameter of the B NPs in water was higher (92 ± 27 nm), which is attributed to the formation of a Stern layer on the nanoparticles in the solution and the different measuring principles of the two methods (Figure 1b). The nanoparticles were coated with polyethylene glycol (5 kDa) by water hydrolysis of silane-PEG chains in the presence of ammonia. The coating did not significantly change the hydrodynamic diameter in water (Figure 1b), but the B-PEG NPs had higher stability in PBS salt buffer (0.1 M, pH 7.4). Uncoated B NPs aggregated in PBS to a hydrodynamic diameter of 220 ± 76 nm (mode value ± half-width of the peak) within 5 min of incubation. The hydrodynamic diameter of the B-PEG NPs increased from 92 ± 27 nm to 106 ± 31 nm within the same period. Coating with polyethyleneglycol also changed the ζ-potential of the B nanoparticles from –30 ± 7.6 mV to –13 ± 8 mV (Figure 1c). 

**Figure 2:**
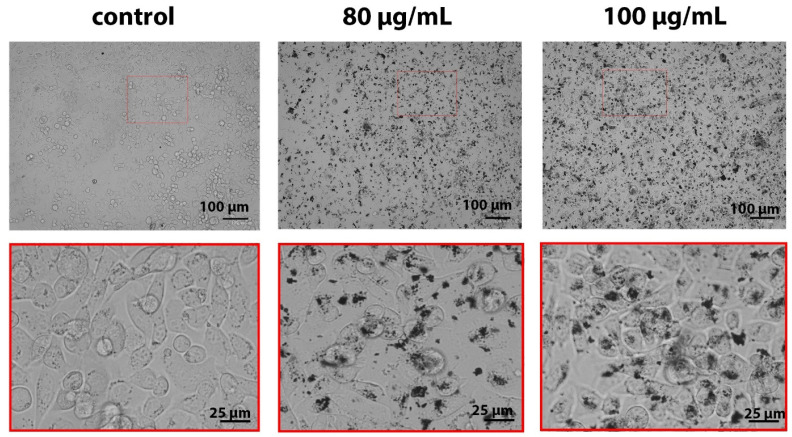
Micrographs of MNNG/Hos osteosarcoma cells after 16 h of incubation with B NPs (80 and 100 µg/mL). Scale bar—100 or 25 µm.

We analyzed the uptake of B NPs by human osteosarcoma cells using optical microscopy. The analysis of micrographs of cells after overnight incubation with B NPs evidences a large quantity of B NPs inside the cells, which is confirmed by dark areas (Figure 2). B NPs are observed in the form of individual particles as well as in the form of aggregates of submicron and micron sizes both on the cell surface and inside cell endosomes. It is worth noting that a repeated washing of cells before irradiation did not lead to the detachment of these B NP aggregates from the cells, and they continued to sit on the cell membrane. Such a character of the aggregation and distribution of B NPs can lead not only to mechanical damage of the cell membrane but also to the initiation of the process of lipid peroxidation of cell membranes under irradiation conditions, which makes a significant contribution to the triggering of apoptosis. Previously, we analyzed the boron content in MNNG/Hos cells using the ICP-MS method, which showed that only half of the applied dose accumulates in cells after 16 h of co-incubation [28].

**Figure 3:**
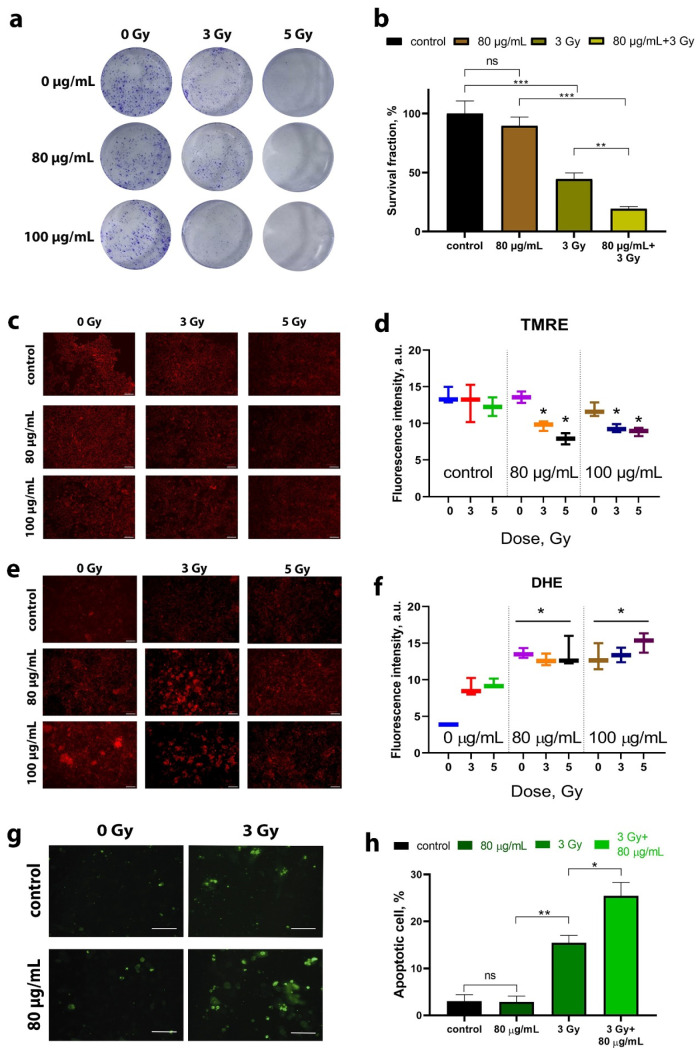
Clonogenic analysis (**a**,**b**), membrane mitochondrial potential (MMP) analysis (**c**,**d**), superoxide radical analysis (**e**,**f**), and apoptosis analysis (**g**,**h**) of the MNNG/Hos human osteosarcoma culture after incubation with the B NPs and proton beam irradiation. Scale bar—100 µm. Data presented as mean ± SD. *p* values were based on Student’s *t* test: * *p* < 0.05, ** *p* < 0.01, *** *p* < 0.001.

A clonogenic assay was used to assess the radiosensitizing effect of B NPs. This test consists in analyzing the ability of cells to undergo mitotic division and colony formation after exposure to ionizing radiation [30]. We found that B NPs themselves do not provide a significant inhibition of colony formation (Figure 3a). On the other hand, irradiation of osteosarcoma cells at a dose of 3 Gy leads to a two-fold decrease in the number of colonies, and at a dose of 5 Gy, colonies are not formed at all (Figure 3b). The combined effect of B NPs at 80 µg/mL and a dose of 3 Gy leads to a four-fold decrease in the number of colonies compared to the non-irradiated control group. Thus, it can be concluded that the combined effect of B NPs and a proton beam has a pronounced inhibitory effect on the mitotic activity of cells.

Next, the mitochondrial membrane potential (MMP) was analyzed after proton beam irradiation of cells in the presence of B NPs (Figure 3c,d). For this purpose, cationic fluorophore tetramethylrodamine (TMRE) was used, which accumulates potential-dependent polarized mitochondria and serves as an indicator of MMP [31]. It was found that proton beam irradiation reduces the MMP for both doses of 3 and 5 Gy. A pretreatment of cells with B NPs at 80 µg/mL does not cause a significant decrease in the TMRE signal, while 100 µg/mL causes a slight decrease in TMRE fluorescence. The combined effect of B NPs and proton irradiation leads to a statistically significant decrease in the level of TMRE fluorescence for B NPs at concentrations of 80 and 100 µg/mL and irradiation doses of 3 and 5 Gy. Thus, one can conclude that the pretreatment of osteosarcoma cells with B NPs is capable of reducing the metabolic activity of irradiated tumor cells via depolarization of mitochondrial membranes. 

Taking into account the recorded violation of MMP during irradiation, we performed a study of the superoxide radical formation, which can be produced directly in the mitochondria as a result of disruption of the respiratory chain [32]. A quantitative analysis was carried out using the superoxide indicator dihydroethidium (DHE) 24 h after proton irradiation (Figure 3e,f). It was found that proton irradiation leads to an increase in the fluorescence values of DHE, regardless of the irradiation dose. Moreover, B NPs themselves at concentrations of 80 μg/mL cause an increase in the level of DHE fluorescence even in the absence of proton irradiation. The combined effect of B NPs (80 μg/mL) and irradiation (3 Gy) leads to a statistically significant increase in the level of DHE (*p* < 0.05). It is worth noting that increasing the dose up to 5 Gy and the concentration of B NPs up to 100 μg/mL did not result in an increase in the fluorescence signal.

Next, we analyzed the number of apoptotic cells 72 h after proton irradiation. For this purpose, the fluorescent dye YO-PRO^®^-1 was employed, which is normally used to identify early apoptotic cells (Figure 3g,h). It was found that B NPs themselves at a concentration of 80 μg/mL do not cause the development of apoptosis. Irradiation at a dose of 3 Gy leads to a significant increase in the number of positive-stained cells by three times (up to 15%) compared to the non-irradiated control. A subsequent analysis 72 h after the irradiation (3 Gy) with B NPs (80 μg/mL) demonstrates a statistically significant increase in the proportion of apoptotic cells (up to 25–30% of the total number of cells). 

**Figure 4:**
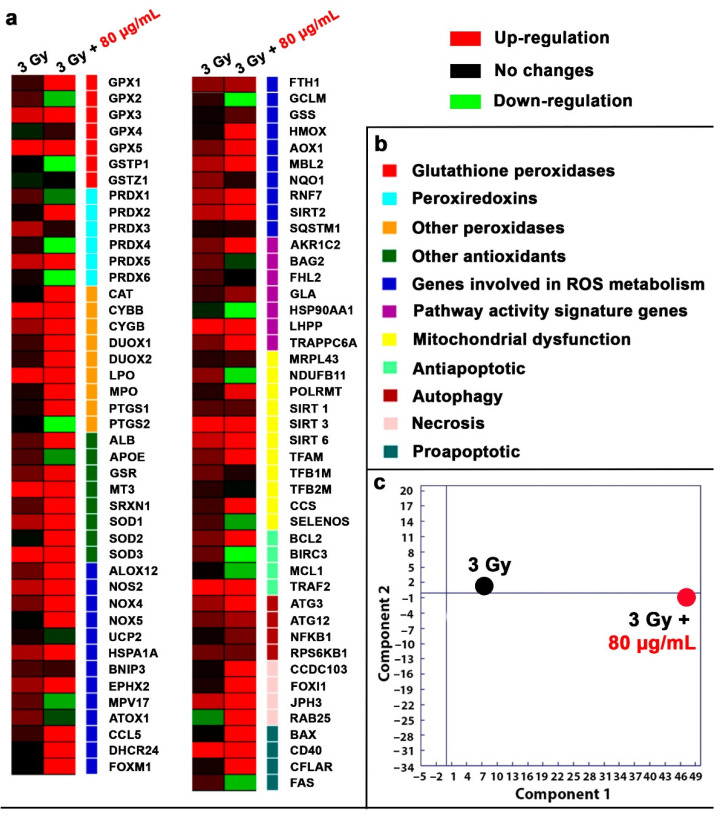
Gene expression in the osteosarcoma MNNG/Hos cells after 24 h of proton beam irradiation with B NPs (80 µg/mL). Data are presented as a heat map (**a**). The intensity scale for standardized gene expression values ranges from −3 (green, decreased expression) to 3 (red, increased expression), with an intermediate intensity of 1:1 (black) corresponding to control values. Cluster groups of genes with the designations of their functions and colors on the heat map (**b**). Principal component analysis (PCA) of qRT-PCR data (**c**).

The analysis of the expression of 87 specially selected genes was carried out using the RT-PCR method (Figure 4). The results of the analysis are presented as a hit map relative to the non-irradiated control, expressed on a logarithmic scale (Figure 4a). The expression of 87 genes responsible for the redox status of cells, anti- and pro-oxidant enzymes, as well as the development of mitochondrial dysfunction, apoptosis, and autophagy was studied (Figure 4b). The heat map shows that proton irradiation at a dose of 3 Gy leads to the activation of only a small part of the selected genes (12 overexpressed genes out of the 87 studied). In particular, overexpression of the GPX3 and GPX5 genes, encoding antioxidant enzymes of the glutathione peroxidase family, was detected. Genes of the glutathione peroxidase family (GPX2, GPX3, GPX4, GPX5) are involved in the antioxidant defense of cells. These enzymes neutralize ROS using reduced glutathione as a substrate for oxidation. The level of GPX gene expression depends on the presence of pro- or antioxidants in the cell and the presence of oxidative stress. Overexpression of the SIRT3 gene, which belongs to a group of genes involved in the development of mitochondrial dysfunction, was also detected. The mitochondrial NAD-dependent deacetylase sirtuin 3 is involved in the regulation of metabolic processes in mitochondria and in the modulation of mitochondrial ROS levels [33]. Overexpression of MT3, SOD1, and SOD3 was detected. Superoxide dismutases (SODs) belong to a group of enzymes that catalyze the reaction of disproportionation of superoxide to hydrogen peroxide and oxygen. The expression of SOD1-3 genes is up-regulated by various factors, including NF-kB, Nrf2, FOXM1, and ROS [34]. Proton beam irradiation induced overexpression of the LPO, CYBB, and CD40 genes, which are responsible for the development of oxidative stress and apoptosis. In this case, the combined effect of B NPs and subsequent irradiation leads to a significant increase in the number of genes that show overexpression (52 genes showing overexpression). In particular, overexpression of genes of the glutathione peroxidase family (GPX1, GPX3, GPX5), genes of the peroxiredoxin family (PRDX2, PRDX5), catalase (CAT), genes of the superoxide dismutase family (SOD1, SOD2, SOD3), genes involved in ROS metabolism (ALOX12, NOS2, NOX4, NOX5, HSPA1A, EPHX2, CCL5, DHCR24, FOXM1), mitochondrial dysfunction genes (SIRT3, SIRT6, and TFAM), necrosis genes (CCDC103, FOXI1, JPH3, RAB25), autophagy genes (ATG3), and proapoptotic genes (BAX, CD40, CFLAR), which encode one of the key enzyme antioxidants, were revealed. A PSA analysis (Figure 4b) clearly demonstrates the pronounced contribution of B NPs under proton irradiation conditions to the overexpression of the studied genes. The combined effect of 80 μg/mL B NPs + 3 Gy induces overexpression of a whole pool of genes that were not activated in response to irradiation, in particular, catalase (CAT), which indirectly confirms a significant increase in the generation of hydrogen peroxide in the cell. All superoxide dismutases (SOD1, SOD2, and SOD3) are activated, which is consistent with the increase in catalase expression, given that the superoxide anion is quickly dismutated by the enzyme to hydrogen peroxide. It is worth noting that SOD2, which is mitochondrial, is also activated. The combined effect activates the expression of almost all genes involved in ROS metabolism (ALOX12, NOS2, NOX4, NOX5, HSPA1A, EPHX2, CCL5, DHCR24, FOXM1). The action of B NPs vs. B NPs combined with proton beam irradiation does not overlap in the three-dimensional principal component analysis (PCA) space, which reliably shows a radiosensitizing effect through an amplification of the gene expression pattern associated with oxidative stress (Figure 4c). Thus, we can say that irradiation of cells in the presence of boron nanoparticles causes the development of radiation-induced oxidative stress. 

## 3. Discussion

Presently, there are two boron compounds that are used clinically: p-boronophenylalanine (BPA) and sodium borocaptate (BSH). BPA is a phenylalanine derivative, and it is transported into cells through an L-type amino acid transporter (LAT1), which is expressed on various cancer cells more than on normal cells [35]. BSH is an anionic carborane derivative with 12 ^10^B atoms that has been previously administered, especially to patients affected by malignant brain tumors [36]. Despite the proven efficiency of boron delivery using these drugs, the issue of ensuring the selectivity of the accumulation of boron-containing drugs and maintaining high doses of boron in the cell remains unresolved. The use of nanotechnology allows us to come closer to finding the solution to this problem. Intracellular delivery of boron in the form of nanoparticles is more effective compared to BPA or BSH, so we can not only provide a local high concentration of boron but also, via additional functionalization by appropriate molecules such as affibodies or DARPins [37], offer a targeted delivery to the tumor. We have shown that human osteosarcoma cells of the MHHG/HOS line effectively absorb B NPs in concentrations sufficient to carry out boron–proton interactions. NPs accumulate in the cytoplasm of cells and are also firmly associated with the cell membrane in the form of aggregates without penetrating into them. Lysosomal localization and accumulation of absorbed boron-containing NPs may lead to the release of boron and the formation of boric acid, which may contribute to cytotoxicity. It was previously shown that boric acid can act as a redox-active agent and contribute to the death of tumor cells [38]. Boric acid and boronic acids can react with various functional groups, such as thiols and amines, allowing the formation of reversible nonionic bonds with enzyme residues [39]. Thus, boron compounds can potentially interact with various metabolites and enzymes, thereby affecting cell proliferation, migration, and metabolic activity. In particular, it was previously shown for the DU-145 cell culture that boric acid causes proliferative inhibition independent of cell death via its effect on the cell cycle and mitochondrial function [40]. It is worth noting that this cell culture was used to confirm the effectiveness of boron–proton interactions in the first experimental confirmation of boron–proton sensitization [17]. Thus, it can be assumed that the efficiency of absorption of boron-containing compounds and the process of their metabolism in the cell can have a significant contribution to the effectiveness of sensitization.

It should be noted that nanoparticles can exhibit surface catalytic properties that are not possible for molecular boron compounds. In particular, we showed that B NPs are able to increase the level of superoxide generation even without irradiation (Figure 3e,f), which may indirectly confirm the induction of certain redox-active processes in the cell by boron-containing nanoparticles. In particular, boron doping of carbon tubes has been used to increase catalytic activity for the oxygen reduction reaction with peroxide production. On the other hand, boric acid formed during the dissolution of nanoparticles is capable of neutralizing hydrogen peroxide, but this reaction practically does not occur at the acidic pH characteristic of late endosomes and lysosomes.

The mitochondrial function is directly related to the state of their polarization. Intact mitochondria maintain a highly charged (internally negative) membrane potential for full functionality [41]. MMP is a key feature of mitochondria because loss of membrane potential accompanies various cellular responses, including uncoupling of oxidative phosphorylation, release of cytochrome c, and initiation of apoptosis [42]. B NPs without proton beam irradiation do not reduce MMP, but proton irradiation leads to a decrease in MMP. It was previously shown that proton irradiation leads to the depolarization of mitochondria and a decrease in MMP due to the formation of radiation-induced nanopores in the membrane [43]. The authors confirmed this effect by visualizing the process of radiation-induced relocation of the TMRE dye from mitochondria into the cytoplasm and then into the extracellular space. A long-term preservation of low MMP in the group subjected to the combined action of B NPs and proton irradiation (more than 24 h after irradiation) shows that such a change can be caused by a radiation-induced formation of ROS, the triggering of lipid peroxidation, or the action of secondary electrons created in the path of protons as a result of ionization, which disrupt the electron transport chain within mitochondria [44].

The gene expression analysis clearly demonstrates that the combined action of proton beam irradiation and B NPs triggers significant oxidative stress, demonstrating a significant increase in the number of overexpressed genes. For example, catalase (CAT gene), which was not overexpressed during irradiation, was hyperactivated. The expression of the CAT gene, an enzyme that catalyzes the breakdown of hydrogen peroxide, is suppressed by the PI3K/Akt/mTor axis [45], and Nrf2 is activated [46]. This suggests that a large amount of hydrogen peroxide is formed in the cell. The source of high levels of hydrogen peroxide may be mitochondrial SOD, the activation of which was also observed. At the same time, we see a statistically significant increase in the level of superoxide anion and depolarization of mitochondrial membranes. These are consistent with the fact that it was previously shown that proton–boron capture therapy increases mitochondrial autophagy in vivo [18]. Thus, we can hypothesize that redox processes associated with mitochondria may make a significant contribution to the boron–proton triggering of apoptosis in cancer cells.

This is the first study that evaluates the effect of B NPs on osteosarcoma cancer cells in vitro, as well as the molecular mechanisms of their bioactivity. Here, we demonstrate that one mechanism of radiosensitization, other than nuclear reactions, may involve increased generation of ROS, changes in cellular redox status, and greater oxidative stress, all of which lead to apoptosis. However, apparently, the radiosensitizing effect of boron-containing compounds in boron–proton interactions is based on a complex mechanism that includes both the generation of alpha particles and redox processes that initiate the development of oxidative stress, leading to apoptosis of cancer cells.

It should be noted that, in our study, we used elemental boron nanoparticles prepared by the technique of femtosecond laser ablation, which was introduced as a powerful tool to finely control the size of NPs formed in ultraclean environments such as deionized water [47]. Previously, this technique demonstrated a high efficiency in the synthesis of a series of nanomaterials for nuclear medicine and other biomedical applications, including Au [48], Bi [10], and TiN [49] nanoparticles. 

## 4. Materials and Methods

### 4.1. Synthesis of Boron Nanoparticles

Boron nanoparticles were synthesized using the method of pulsed laser ablation in liquids [29]. A crystalline boron target was vertically fixed inside a glass cuvette containing 50 mL of deionized water (18.2 MΩ/cm). The laser beam (3 mm diameter) from a Yb:KGW femtosecond laser (TETA-10, Avesta, Moscow, Russia; 1030 nm, 270 fs, 30 µJ, 200 kHz) was focused on the target surface using an F-theta lens with a 100 mm working distance. The thickness of the liquid layer between the inner glass wall and the boron target was 2.5 mm. A galvanometric system (LScan-10, Ateko-TM, Moscow, Russia) was used to move the laser beam across the target surface at a speed of 4 m/s, following a self-enclosed spiral scanning pattern (3 mm outer diameter). The duration of the ablation procedure was 60 min. Large B NPs (>150 nm) were removed from the solution by centrifugation at 13,000× *g* for 1 min.

To coat the boron nanoparticles with polyethylene glycol, 1 mg of B NPs was dispersed in 1 mL of 95% ethanol. Subsequently, 1 μL of 30% NH₄OH and 10 μg of m-silane-PEG (5 kDa) were added to the solution, and the reaction proceeded overnight at room temperature. The coated B-PEG NPs were then washed twice with ethanol and transferred to distilled water by centrifugation at 15,000× *g* for 15 min. 

### 4.2. NP Characterization

The hydrodynamic size of the synthesized B NPs was measured using dynamic light scattering (DLS) with a Zetasizer Nano ZS (Malvern Instruments Ltd., Worcestershire, UK). Size analysis measurements were conducted in distilled water (pH 6) and phosphate-buffered saline (pH 7.4). The hydrodynamic diameters are reported as mode values ± half-width of the peak of the number-weighted size distributions. Smoluchowski approximation was applied for the calculation of the ζ-potential, which was measured in a 10 mM NaCl solution (pH 6).

High-resolution transmission electron microscopy (Jeol JEM-2100 microscope, Tokyo, Japan) was used to obtain images of the B NPs. The TEM was equipped with a 200 kV field emission gun and had a point resolution of 0.19 nm. Samples for TEM measurements were prepared by dropping 1 µL of the colloidal solution onto a carbon grid, which was then dried under ambient conditions. The size distribution of the boron NPs was determined by analyzing the TEM images using ImageJ software 1.54j with a circle fit approximation. The final distribution was based on measurements of 150 boron NP diameters.

### 4.3. Cell Line

Human osteosarcoma cells of the MNNG/Hos line were used to analyze proton-enhanced radiosensitization by B-PEG NPs. The MNNG/HOS cells were cultured in DMEM/F-12 medium (1:1, PanEco, Moscow, Russia) supplemented with 10% fetal bovine serum (FBS) (HyClone, Logan, UT, USA), 2 mM L-glutamine (PanEco, Moscow, Russia), 100 U/mL penicillin (PanEco, Moscow, Russia), and 100 µg/mL streptomycin (PanEco, Moscow, Russia). The cells were cultivated at 37 °C in a humidified atmosphere containing 5% CO_2_. When the cells reached a sub-confluent state, they were treated with a 0.25% trypsin-EDTA solution (PanEco, Moscow, Russia) and transferred to new culture flacks at a 1:3 ratio. 

### 4.4. Cellular Uptake of Nanoparticles 

Cellular uptake of B-PEG NPs was analyzed using bright-field microscopy. Cells were seeded in 35 mm Petri dishes with a central hole (Ibidi, Madison, WI, USA) at a density of 25 ×10^3^ per cm^2^ and allowed to attach. B-PEG NPs at various concentrations of boron (80–100 µg/mL) were added to the cells and incubated overnight. After 16 h, the cells were washed three times with Hanks’ solution and then photographed using a Biorad ZOE imager (Biorad, Hercules, CA, USA) in bright-field mode. 

### 4.5. Proton Beam Irradiation

Cell irradiation was performed on the proton therapy complex “Prometheus”. To irradiate cells in the Bragg peak, a 50 × 60 mm irradiation field was formed. The energy of the extracted proton beam was 160.5 MeV. To place a monolayer of cells directly in the Bragg peak, a 151.8 mm polymethylmethacrylate (PMMA) moderator was placed in the beam path. Dose uniformity and field size were monitored using an EBT3 radiometric film, and the absorbed dose was monitored using a PTW Unidos webline electrometer with a PTW PinPoint 3D Chamber 31022 ionization chamber. For dosimetry in the Bragg peak, an additional PTW Bragg Peak Chamber 34073 ionization chamber was used. At the level of 95% isodose, the homogeneity was higher than 98%. 

For proton beam irradiation, cells were seeded into 12.5 cm^2^ culture flasks (JetBiofil, Guangzhou, China) or 8-well plates (SPL LifeScience, Pocheon-si, Republic of Korea) at a density of 25 × 10^3^ per cm^2^. Before irradiation of the flasks and plates, they were filled with culture medium containing no serum, and the lids were fixed with parafilm. The culture flasks were kept in a thermostat at 37 °C before proton beam irradiation. Irradiation of a vertically positioned flask was carried out in the scanning beam mode (pencil-beam proton therapy).

### 4.6. Clonogenic Assay

After proton beam irradiation, MNNG/Hos cells were seeded in 6-well plates (SPL LifeScience, Pocheon-si, Republic of Korea) at a concentration of 1000 cells per well in DMEM/F12 culture medium supplemented with 10% FBS. The cells were cultured at 37 °C in a humidified atmosphere containing 5% CO_2_. Once colony formation was complete in the control group (after 7–8 days), the cells were washed three times with PBS buffer, fixed in a 4% paraformaldehyde solution (Sigma Aldrich, Burlington, MA, USA), and stained with 0.1% crystal violet solution (Sigma Aldrich, Burlington, MA, USA). Cell clusters of more than 50 cells were considered as a single colony. Colonies were counted manually using a magnifying glass.

### 4.7. Apoptosis Analysis

Quantitative analysis of apoptotic cells was conducted using fluorescence microscopy with the YO-PRO-1 dye (Biotium, Landing Pkwy, Fremont, CA, USA). YO-PRO-1 is a green fluorescent, cell-impermeant, high-affinity carbocyanine monomeric nucleic acid stain that is specifically used to identify apoptotic cells. Cells were seeded in 12.5 cm^2^ flasks at a density of 25 × 10^3^ per cm^2^ and allowed to attach, and then B-PEG NPs at 80 µg/mL concentration were added and incubated overnight. The cells were irradiated at a dose of 3 Gy at Bragg peak, and 4 h after proton irradiation, they were seeded into 96-well plates. After 24 h of irradiation, the cells were washed three times with PBS buffer and then stained with the YO-PRO-1 dye at a concentration of 10 µM for 20 min in the dark. Green fluorescence-positive cells were detected by a Biorad ZOE imager (Biorad, Hercules, CA, USA). For quantitative analysis, 3 cell areas were analyzed in 5 separate wells.

### 4.8. Detection of Superoxide Radicals

Superoxide anion production was measured using the fluorescent dye dihydroethidium (DHE) (Invitrogen, Carlsbad, San Diego, CA, USA). The DHE fluorescence assay detects nuclear O_2_^●−^ through a chemical reaction in which superoxide oxidizes DHE to ethidium, which exhibits red fluorescence. Cells were seeded on 8-well plates (SPL LifeScience, Pocheon-si, Republic of Korea) at 2.5 × 10^3^ cells per cm^2^ density in DMEM/F12 culture medium supplemented with 10% FBS. The cells were pretreated with B-PEG NPs (80 and 100 µg/mL) for 16 h. Before proton beam irradiation, the cells were washed three times with PBS buffer, and the medium was replaced with fresh one. After 24 h of irradiation, the culture medium was replaced with a 2 µM DHE solution. The cells were incubated for 30 min at 37 °C in a humidified atmosphere containing 5% CO_2_ in the darkness. DHE fluorescence in the cells was detected by a Biorad ZOE imager.

### 4.9. Analysis of Mitochondrial Membrane Potential 

Mitochondrial membrane potential (MMP) was determined using the tetramethylrhodamine ethyl ester (TMRE) dye (ThermoFisher, Carlsbad, CA, USA) via fluorescence microscopy. TMRE accumulates in the mitochondrial membrane in a potential-dependent manner. Cells were seeded into 8-well tissue culture plates (SPL, LifeScience, Republic of Korea) at a density of 2.5 × 10^3^ cells/cm^2^ and cultured in a CO_2_ incubator at 37 °C for 24 h with different concentrations of B-PEG NPs. The cells were then preincubated with 0.5 μM TMRE in Hanks’ solution in a CO_2_ incubator at 37 °C for 20 min. Next, the cells were washed twice with Hanks’ solution and analyzed using a BioRad ZOE fluorescent imager.

### 4.10. Real-Time PCR

One million cells were collected by trypsinization and transferred to 500 µL of EverFresh RNA solution (Sileks, Moscow, Russia). The cell samples were stored at −20 °C until the experiments. An ExtractRNA kit was used for mRNA isolation according to the manufacturer’s protocol (Evrogen, Moscow, Russia). Briefly, the frozen cell suspension was homogenized using a pestle in a test tube, after which a lysing buffer was added for 10 min. Then, the cell suspension was centrifuged at 10,000× *g* for 5 min, and the supernatant was collected and mixed with chloroform. Then, samples were centrifuged, and the supernatant was subsequently washed with 100% isopropanol and 96% ethanol. The resulting RNA precipitate was dried at room temperature for 7 min. The genomic DNA was removed by incubation with DNAse solution (Eurogene, Moscow, Russia). All incubation steps were performed on ice.

cDNA synthesis was performed with a reverse transcription kit with oligo(dT) primer supplied by Sileks (Moscow, Russia). The protocol uses M-MLV reverse transcriptase, which is a product of the pol gene of the Moloney Murine Leukemia Virus (MMLV). A mixture of primers and RNA matrix with water was incubated for 5 min at 70 °C. Then, M-MLV reverse transcriptase was introduced in a buffer containing a dNTP mixture, and the solution was incubated for 1 h at 37 °C and 10 min at 70 °C. The derived cDNA was used as a template for real-time PCR. The reaction was conducted on a CFX-96 thermal cycler (BioRad, Hercules, CA, USA) using a 5X qPCRmix-HS solution, having HS Taq DNA polymerase and SybrGreen I dye. 

The expression levels of 96 genes associated with redox status and the antioxidant system were measured (Supplementary Materials: Table S1). Gene selection was based on the database at http://www.sabiosciences.com/ (accessed on 13 May 2021) for PCR profiling of different biological processes. The transcription level was normalized using the expression of housekeeping genes encoding β-actin, RPLP0 (ribosomal protein, large, P0), and GAPDH (glyceraldehyde-3-phosphate dehydrogenase). Gene-specific primers were picked using Primer Express software 6.0 (Applied Biosystems, Foster City, CA, USA). Each measurement was performed in duplicate (internal replication) and averaged from two independent samples. Samples without reverse transcription were used as controls. Analysis of the expression data was performed using Genesis software 9.07.

### 4.11. Statistical Analysis

Data are presented as mean ± standard deviation, if not stated otherwise. Statistical analysis was carried out using two-tailed Student’s *t* test. *p* values of less than 0.001 (***), 0.01 (**), and 0.05 (*) were considered to be significant. The statistical analysis was performed using the GraphPrism Program, 8.0.1 version.

## 5. Conclusions

We evaluated possible molecular mechanisms of B NP radiosensitizing under proton beam irradiation. We associate the effects of radiosensitization with a violation of the cellular redox balance and the development of pronounced oxidative stress through extended ROS generation in the presence of B NPs. In particular, we found that irradiation of the cells by a 160.5 MeV proton beam at a dose of 3 Gy in the presence of 80 and 100 µg/mL of B NPs led to a significant increase in the level of the superoxide radical, as well as a drop in the membrane mitochondrial potential. We also observed the overexpression of a large number of genes responsible for oxidative stress, the development of apoptosis, and the antioxidant defense system of the cell, which was not observed in the absence of B NPs. Redox reactions involving boron-containing compounds, including B NPs, are poorly understood and can help uncover the molecular mechanisms of their radiosensitizing effect under proton irradiation, and the development of new effective intracellular boron delivery systems, such as nanoparticles, will bring boron–proton therapy closer to preclinical and clinical studies.

## Data Availability

Data are available upon reasonable request from the corresponding author.

## Supplementary Materials

The following supporting information can be downloaded at https://www.mdpi.com/article/10.3390/molecules29163936/s1, Table S1: Estimation of gene expression in cultured cells by the method of real-time (RT) PCR and primers used in the study.
