# Supplementary material for: Boron Nanoparticle-Enhanced Proton Therapy: Molecular Mechanisms of Tumor Cell Sensitization
Source: Molecules. 2024 Aug 21;29(16):3936. doi: 10.3390/molecules29163936 (PMC11357428; doi:10.3390/molecules29163936)
Supplement: Supplementary file 1 [file molecules-29-03936-s001.zip › molecules-3139793-supplementary.pdf]

Boron Nanoparticle-Enhanced Proton Therapy: Molecular Mechanisms of Tumor Cell Sensitization

Anton L. Popov [1,2], Danil D. Kolmanovich [1,2], Nikita N. Chukavin [2], Ivan V. Zelepukin [3,4], Gleb V. Tikhonowski [5], Andrei I. Pastukhov [6], Anton A. Popov [5], Alexander E. Shemyakov [1], Sergey M. Klimentov [5], Vladimir A. Ryabov [1], Sergey M. Deyev [3,5,7,8,9], Irina N. Zavestovskaya [1,5,7,*] and Andrei V. Kabashin [6,*]

1 P. N. Lebedev Physical Institute of the Russian Academy of Sciences, Leninsky Prospect 53, Moscow 119991, Russia; a.popov@lebedev.ru (A.L.P.); kdd100996@mail.ru (D.D.K.); shemyakovae@lebedev.ru (A.E.S.); ryabov@lebedev.ru (V.A.R.)
2 Institute of Theoretical and Experimental Biophysics, Russian Academy of Sciences, 3 Institutskaya St., Pushchino 142290, Russia; chukavinnik@gmail.com
3 Shemyakin-Ovchinnikov Institute of Bioorganic Chemistry, Russian Academy of Sciences, Moscow 117997, Russia; ivan.zelepukin@gmail.com (I.V.Z.); biomem@mail.ru (S.M.D.)
4 Department of Medicinal Chemistry, Uppsala University, 75310 Uppsala, Sweden
5 Institute of Engineering Physics for Biomedicine (PhysBio), National Research Nuclear University MEPhI, Kashirskoe Shosse 31, Moscow 115409, Russia; gtikhonowski@gmail.com (G.V.T.); aapopov1@mephi.ru (A.A.P.); smklimentov@mephi.ru (S.M.K.)
6 LP3, CNRS, Aix-Marseille University, 13288 Marseille, France; andrei.pastukhov@etu.univ-amu.fr
7 National Research Center "Kurchatov Institute", Academician Kurchatov Square 1, Moscow 123182, Russia
8 "Biomarker" Research Laboratory, Institute of Fundamental Medicine and Biology, Kazan Federal University, 18 Kremlyovskaya St., Kazan 420008, Russia
9 Institute of Molecular Theranostics, Sechenov University, Moscow 119991, Russia
* Correspondence: zavestovskayain@lebedev.ru (I.N.Z.); andrei.kabashin@univ-amu.fr (A.V.K.)

Table S1. The estimation of the genes expression in cultured cells by the method of the real time (RT) PCR, and the primers used in the study.

Function	Description	GeneBank	Symbol	Forward 5'-3'	Rewerse 5'-3'
Glutathione Peroxidases (GPx)	Glutathione peroxidase 1	NM_000581	GPX1	CCTCCCCTTACAGTGCTTGTTC	GCACACATGGCGCAATTG
	Glutathione peroxidase 2 (gastrointestinal)	NM_002083	GPX2	CCGATCCCAAGCTCATCATT	TCTCAAAGTTCCAGGCCACAT
	Glutathione peroxidase 3 (plasma)	NM_002084	GPX3	CATCCCCTTCAAGCAGTATGCT	GCCCGTCAGGCCTCAGTAG
	Glutathione peroxidase 4 (phospholipid hydroperoxidase)	NM_002085	GPX4	CCGATACGCTGAGTGTGGTTT	GCTCCTGCTTCCCGAACTG
	Glutathione peroxidase 5 (epididymal androgen-related protein)	NM_001509	GPX5	TCACCACACTCTCTTCCTGCAT	AGAGTGGGAATTCTGGCAGTATG

	Glutathione S-transferase pi 1	NM_000852	GSTP1	CAGGAGGGCTCACTCAAAGC	GTGAGGTCTCCGTCCTGGAA
	Glutathione transferase zeta 1	NM_001513	GSTZ1	CCCAGAACGCCATCACTTG	TGCCCGCTGTGCTCTGT
Peroxiredoxins (TPx)	Peroxiredoxin 1	NM_002574	PRDX1	CTGGGACCCATGAACATTCC	AAGACCCCATAATCCTGAGCAA
	Peroxiredoxin 2	NM_005809	PRDX2	TCCTTCGCCAGATCACTGTTAA	CAGCCGCAGAGCCTCATC
	Peroxiredoxin 3	NM_006793	PRDX3	GCATTTGAGCGTCAACGATCT	TCACCAAGCGGAGGGTTTC
	Peroxiredoxin 4	NM_006406	PRDX4	GAGGCATCCCGGGTATCG	GGCTTGGAAATCTTCGCTTTG
	Peroxiredoxin 5	NM_181652	PRDX5	AGATGATTCGCTGGTGTCCAT	ACTATGCCATCCTGTACCACCA T
	Peroxiredoxin 6	NM_004905	PRDX6	GGCCGCATCCGTTTCC	CCCGAGGGTGGGAGAAGA
Other Peroxidases	Catalase	NM_001752	CAT	CAGGGCATCAAAAACCTTTCTG	CGGATGCCATAGTCAGGATCTT
	Cytochrome b-245, beta polypeptide	NM_000397	CYBB	CCTTTGAGTGGTTTGCAGATCTG	AGCCGGCATTGTTCCTTTC
	Cytoglobin	NM_134268	CYGB	GCAGCACCTCGAGCAGAAG	CCTTGGCACCCAGAAATGG
	Dual oxidase 1	NM_175940	DUOX1	TGAGCGGCACTTCCAGAAG	GACGGCCAAAGTGGGTGAT
	Dual oxidase 2	NM_014080	DUOX2	CCTTCGAGCCCTTCTTCAACT	CAGCTGAACACCCCGATCTT
	Lactoperoxidase	NM_006151	LPO	CAAGCTTTTCCAGCCAACTCA	CCGGCAACGCTGTGTGT
	Myeloperoxidase	NM_000250	MPO	CCTGAAATTGGCGAGGAAACT	GCCGCCCATCCAGATGT
	Prostaglandin-endoperoxide synthase 1	NM_000962	PTGS1	TGTTCGGTGTCCAGTTCCAATA	TGCCAGTGGTAGAGATGGTTGA
	Prostaglandin-endoperoxide synthase 2	NM_000963	PTGS2	AATTGCTGGCAGGGTTGCT	GGTCAATGGAAGCCTGTGATAC TT
Other Antioxidants	Albumin	NM_000477	ALB	TGAGAAAACGCCAGTAAGTGACA	GAAAAGCATGGTCGCCTGTT
	Apolipoprotein E	NM_000041	APOE	CTGCGTTGCTGGTCACATTC	CTCTGTCTCCACCGCTTGCT
	Glutathione reductase	NM_000637	GSR	TGCAGGGACTTGGGTGTGA	GCCTTCGTTGCTCCCATCT
	Metallothionein 3	NM_005954	MT3	AGTGCGAGGGATGCAAATG	GCCTTTGCACACACAGTCCTT
	Sulfiredoxin 1	NM_080725	SRXN1	TGCTGTATCCCCAAGAATCATG	GCTAGTTTGGCCCTTCCTCTTC
	Superoxide dismutase 1, soluble	NM_000454	SOD1	TGGTGTGGCCGATGTGTCT	GTGCGGCCAATGATGCA
	Superoxide dismutase 2, mitochondrial	NM_000636	SOD2	TCCGCAGAAAGGAACATTAAGG	TGACCTCCATTCTTTGCTCTCA
	Superoxide dismutase 3, extracellular	NM_003102	SOD3	GCGGAGCCCAACTCTGACT	TGCCAGATCTCCGTGACCTT
Genes Involved in Reactive Oxygen Species (ROS) Metabolism	Arachidonate 12-lipoxygenase	NM_000697	ALOX1 2	CCACCCACCACCAAGGAA	TGCCGGACATCAGGTAGTGA
	Nitric oxide synthase 2, inducible	NM_000625	NOS2	CCGCATGACCTTGGTGTTT	TCCAGCATCTCCTCCTGGTAGA
	NADPH oxidase 4	NM_016931	NOX4	AAGAGCCCAGATTCCAAGCTAATT	CGGCACAGTACAGGCACAAA
	NADPH oxidase, EF-hand calcium binding domain 5	NM_024505	NOX5	AGGCACCAGAAAAGAAAGCATAC T	ATGTTGTCTTGGACACCTTCGAT

	Uncoupling protein 2 (mitochondrial, proton carrier)	NM_003355	UCP2	CAGTTCTACACCAAGGGCTCTGA	CCTGTGGTGCTGCCTGCTA
	Aldehyde oxidase 1	NM_001159	AOX1	GGTGTTCCGTGTTTTTCGCTAT	GGTCCATGCAGGCCTCTCT
	BCL2/adenovirus E1B 19kDa interacting protein 3	NM_004052	BNIP3	TCCATCTCTGCTGCTCTCTCATT	AGGTTGTCAGACGCCTTCCA
	Epoxide hydrolase 2, cytoplasmic	NM_001979	EPHX2	AACTGGGCCTCTCTCAAGCA	AGCCATGTACCACACCAGCAT
	MpV17 mitochondrial inner membrane protein	NM_002437	MPV17	TCTATGGCCTGCTGTGCAGTT	GGACAACGGCCAACCTGTA
	ATX1 antioxidant protein 1 homolog (yeast)	NM_004045	ATOX1	TGCTTGCAACCCTGAAGAAA	GGACCAGGCCCCTGCTA
	Chemokine (C-C motif) ligand 5	NM_002985	CCL5	TGCATCTGCCTCCCCATATT	AGTGGGCGGGCAATGTAG
	24-dehydrocholesterol reductase	NM_014762	DHCR24	CATGCTGGTGCCCATGAAG	GACGTGGATGTCGTTTTGGAA
	Forkhead box M1	NM_021953	FOXM1	AGGAAACGCTGCCCATCTC	CGTGAGCCTCCAGGATTCAG
	Ferritin, heavy polypeptide 1	NM_002032	FTH1	CTGGCTTGGCGGAATATCTCT	GCCCGAGGCTTAGCTTTCAT
	Glutamate-cysteine ligase, modifier subunit	NM_002061	GCLM	CCGCCTGCGGAAGAAGT	CATTCAAGGTTTTTGGATACAATCA
	Glutathione synthetase	NM_000178	GSS	GCAGGAAAAGACACTCGTGATG	CATGCTCGATGGCTTTGGT
	Heme oxygenase (decycling) 1	NM_002133	HMOX1	TCCGATGGGTCCTTACACTCA	GCCTGCATTCACATGGCATA
	Heat shock 70kDa protein 1A	NM_005345	HSPA1A	GCTGATTGGCCGCAAGTT	TGGAAAGGCCAGTGCTTCAT
	Mannose-binding lectin (protein C) 2, soluble	NM_000242	MBL2	AGTGAAGGCCTTGTGTGTCAAGT	TCCATTCTCTGCAGCATTCCT
	NAD(P)H dehydrogenase, quinone 1	NM_000903	NQO1	CAGCAGACGCCCGAATTC	TGGTGTCTCATCCCAAATATTCTC
	Ring finger protein 7	NM_014245	RNF7	AAAGGAAAGAGCTCCAAATTGAATC	CATAAGCATGCAAAAAGTTCTCTGA
	Sirtuin 2	NM_012237	SIRT2	GCTGGAACAGGAGGACTTGGT	TGGCGCTGACGCAGTGT
	Sequestosome 1	NM_003900	SQSTM1	GGAAGGTGAAACACGGACACTT	ACGTGGGCTCCAGTTTCCT
Pathway Activity Signature Genes	Aldo-keto reductase family 1	NM_001354	AKR1C2	GATTGCCCTGCGCTACCA	TGTCTGATGCGCTGCTCATT
	BCL2-associated athanogene 2	NM_004282	BAG2	CTCACCGTTGAAGTGTCAGTAGAAA	ATCAATAATCCTTGTGGCATGCT

	Four and a half LIM domains 2	NM_001450	FHL2	CCTGCAGGAAGCAGCTGTCT	AGTTCAGGCAGTAGGCAAAGTCA
	Galactosidase, alpha	NM_000169	GLA	GGATGGCTCCCCAAAGAGAT	GGCGAATCCCATGAGGAAA
	Heat shock protein 90kDa alpha (cytosolic), class A member 1	NM_001017963	HSP90AA1	TTGGCAGTGAAGCATTTTTCAG	GAGCACGTCGTGGGACAAA
	Phospholysine phosphohistidine inorganic pyrophosphate phosphatase	NM_022126	LHPP	TGCGCACCGGGAAGTT	CACGTACCCATCAGCCTTCA
	Trafficking protein particle complex 6A	NM_024108	TRAPPC6A	GGTGTTCCAGAAGCAGATGGA	AGCTGTTGTCTTGCAGGACGTA
Mitochondrial dysfunction	Mitochondrial ribosomal protein L43	NM_176794	MRPL43	CAGTTGCACCGCAGATCCT	GGAAGATCGGATGACTGAACTGA
	NADH dehydrogenase (ubiquinone) 1 beta subcomplex, 11, 17.3kDa	NM_019056	NDUFB11	GCAGCACCTTTGTGGCCTAT	TCCCATCCCACGCTCTTG
	Polymerase (RNA) mitochondrial (DNA directed)	NM_005035	POLRMT	CACAGGTGCTGGAAGGTTTCA	CCGTACACCACCGTCATCAC
	Sirtuin 1	NM_012238	SIRT1	TGAGCCTGATGTTCCAGAGAGA	AGCTTCATTAATTGCCTCTTGATCAT
	Sirtuin 3	NM_012239	SIRT3	CCAGTGGCATTCCAGACTTCA	GATCGTACTGCTGGAGGTTGCT
	Transcription factor B1, mitochondrial	NM_016020	TFB1M	GCCATCGAGGGCTCAGAA	CAGCCTGCCCGTGCTTT
	Transcription factor B2, mitochondrial	NM_022366	TFB2M	AAGGCGTCTAAGGCCAGCTT	TTTGCGCCAGGGTCTCA
	Copper chaperone for superoxide dismutase	NM_005125	CCS	GCCGCGCCATCTTCAG	ATCAGGCTGCGGCCAAT
	Selenoprotein P, plasma, 1	NM_203472	SELENOS	CTGAAACGGAAATCGGACAGA	CGCCTCCTTCACCAGACAAC
Anti Apoptotic	B-cell CLL/lymphoma 2	NM_000633.2	BCL2	CTGGGATGCCTTTGTGGAACT	AGACAGCCAGGAGAAATCAAACAG
	aculoviral IAP repeat containing 3	NM_001165.4	BIRC3	GGACAGGAGTTCATCCGTCAAG	TCTCCTGGGCTGTCTGATGTG
	myeloid cell leukemia 1	NM_021960.4	MCL1	CACGAGACGGCCTTCCAA	CACTCGAGACAACGATTTCACATC
	TNF receptor-associated factor 2	NM_021138.3	TRAF2	GGCCGTCTGTCCCAGTGAT	TTCGTGGCAGCTCTCGTATTC
Autophagy	autophagy related 3	NM_022488.4	ATG3	CCATTGAAAATCACCCTCATCTG	CACCTCAGCATGCCTGCAT
	autophagy related 12	NM_004707.3	ATG12	CCCGGGAACAGAGGAACCT	GGAGTGTCTCCCACAGCCTTT
	nuclear factor of kappa light polypeptide gene enhancer in B-cells 1	NM_003998.3	NFKB1	GGCTACACCGAAGCAATTGAAG	CAGCGAGTGGGCCTGAGA
	ribosomal protein S6 kinase, 70kDa, polypeptide 1	NM_003161.3	RPS6KB1	TGGCATAGAGCAGATGGATGTG	AGAGTTCGGCTGTCGTATTGGA

Necrosis	coiled-coil domain containing 103	NM_213607.2	CCDC103	GCTGCAAGGGCTTGTTTCAG	GCCCCTCCTTCACGGATCT
	forkhead box I1	NM_012188.4	FOXI1	CGCCTCACTCTCAGCCAGAT	CCGGCCTTGCTCTTGTTGTA
	junctophilin 3	NM_020655.3	JPH3	CCAGGATCACTGCCAAAGAGTT	CGCTTCGGCCTCTGGTACT
	RAB25, member RAS oncogene family	NM_020387.2	RAB25	TGTCTTCAAGGTGGTGCTGATC	CGCGTGAATCGGGAGAGTAG
Pro apoptotic	BCL2-associated X protein	NM_004324.3	BAX	GTGGCAGCTGACATGTTTTCTG	GCAAAGTAGAAAAGGGCGACAA
	CD40 molecule, TNF receptor superfamily member 5	NM_001250.4	CD40	ACACTGCCACCAGCACAAATACT	CTGTTTCTGAGGTGCCCTTCTG
	CASP8 and FADD-like apoptosis regulator	NM_003879.5	CFLAR	GTGTGTATGGTGTGGATCAGACTCA	GGCATGAATCTCCCATGAACA
	Fas cell surface death receptor	NM_000043.4	FAS	GAATCATCAAGGAATGCACACTCA	AAAGCCACCCCAAGTTAGATCTG
	Tumor necrosis factor receptor superfamily, member 10a	NM_003844.3	TNFRSF1	CTGGCGCTTGGGTCTCCTA	TGCGTTGCTCAGAATCTCGTT
House keeping	glyceraldehyde-3-phosphate dehydrogenase	NM_002046.5	GAPDH	GTGGAAGGACTCATGACCACAGT	GCCATCACGCCACAGTTTC
	ribosomal protein, large, P0	NM_001002.3	RPLP0	ATGCAGCAGATCCGCATGT	TTGCGCATCATGGTGTTCTT
	beta-actin	XM_006715764.1	Actin	TCGTGCGTGACATTAAGGAGAA	AGCAGCCGTGGCCATCT
